# Correction: A dynamic bactofilin cytoskeleton cooperates with an M23 endopeptidase to control bacterial morphogenesis

**DOI:** 10.7554/eLife.101019

**Published:** 2024-07-04

**Authors:** Sebastian Pöhl, Manuel Osorio-Valeriano, Emöke Cserti, Jannik Harberding, Rogelio Hernandez-Tamayo, Jacob Biboy, Patrick Sobetzko, Waldemar Vollmer, Peter L Graumann, Martin Thanbichler

**Keywords:** Other

Pöhl S, Osorio-Valeriano M, Cserti E, Harberding J, Hernandez-Tamayo R, Biboy J, Sobetzko P, Vollmer W, Graumann PL, Thanbichler M. 2024. A dynamic bactofilin cytoskeleton cooperates with an M23 endopeptidase to control bacterial morphogenesis. *eLife* **12**:RP86577. doi: 10.7554/eLife.86577.

Published 31 January 2024

Going through our publication again, we identified an error in Figure 1C, which shows example images of the *H. neptunium* wild type and the three bactofilin mutants analysed in the study. The first reviewed preprint (v1) still contained a correct version of this panel. However, during the revision of our manuscript after peer review, Figure 1 underwent significant modifications. The preparation of the revised figure involved multiple changes in the arrangement of the elements in Figure 1C, which were in part realized by copying images between different graphic files and/or by re-inserting them again from the source folders. In this process, the images of Δ*bacD* and Δ*badAD* cells shown in the upper row were accidentally duplicated and one of the duplicated images was additionally misplaced in a wrong column. Unfortunately, this error remained unnoticed at later stages of the review and publication process. We apologize for any confusion this issue may have caused.

We have now re-inserted the proper images from reviewed preprint v1, but again only show two instead of the initial four example images of Δ*bacAD* cells to avoid redundancy.

The corrected Figure 1 is shown here:

**Figure 1:**
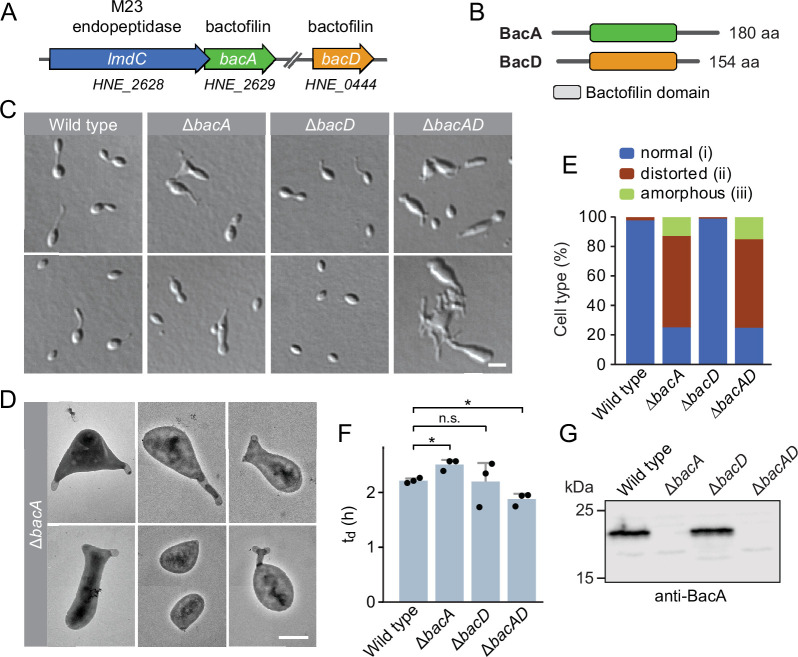


The originally published Figure 1 is shown for reference:

**Figure 2:**
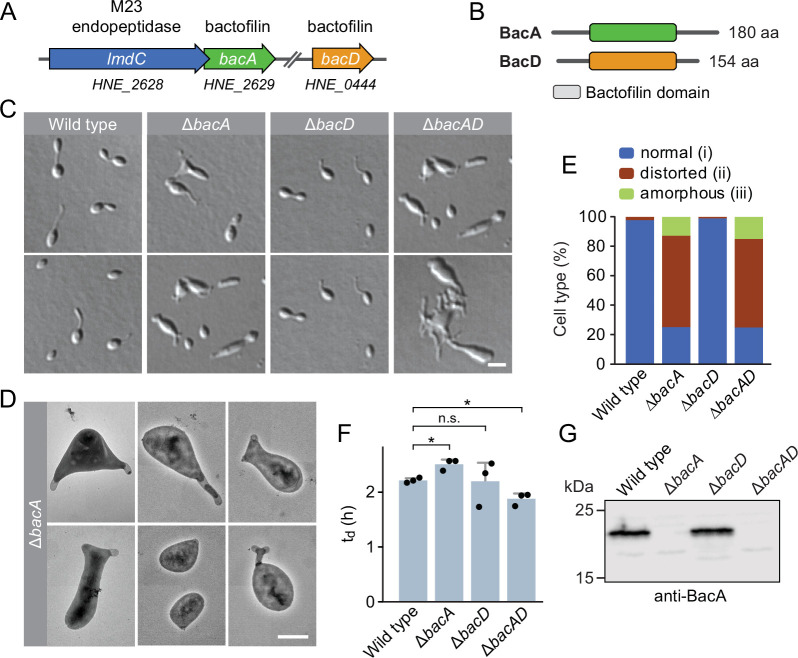


The article has been corrected accordingly.

