# Supplementary figures and images for: A dynamic bactofilin cytoskeleton cooperates with an M23 endopeptidase to control bacterial morphogenesis
Source: eLife. 2024 Jan 31;12:RP86577. doi: 10.7554/eLife.86577 (PMC10945521; doi:10.7554/eLife.86577)

Wild type  
 $\Delta bacA$   
 $\Delta bacD$   
 $\Delta bacAD$

kDa

25 -

15 -

anti-BacA

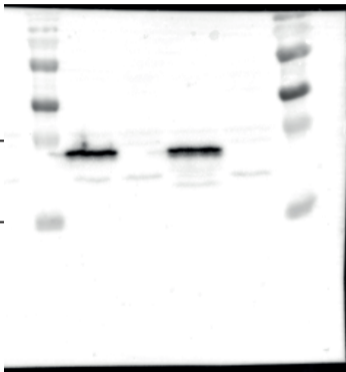

Supplement: Figure 1—source data 1. [file elife-86577-fig1-data1.zip › Figure 1G-source data 1.pdf]

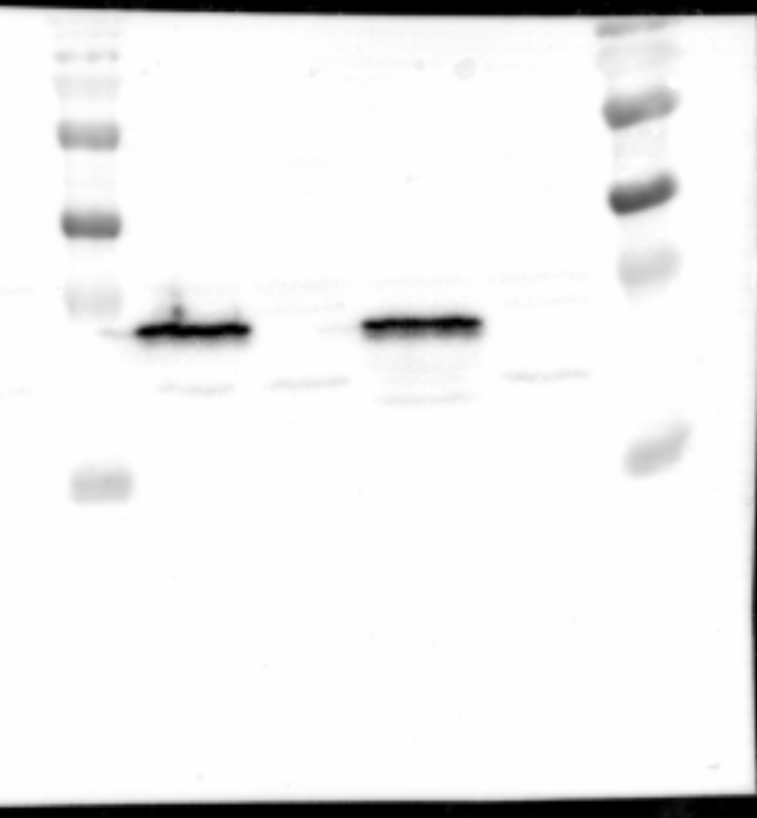

Supplement: Figure 1—source data 1. [file elife-86577-fig1-data1.zip › Figure 1G-source data 1.tif]

Wild type  
 $\Delta$ bacA  
-bacA  
+bacA

kDa

25

15

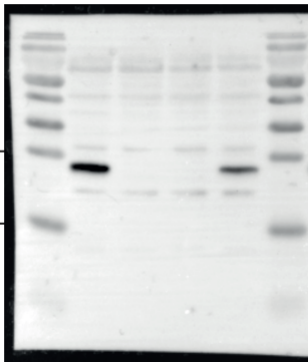

anti-BacA

Supplement: Figure 1—figure supplement 2—source data 1. [file elife-86577-fig1-figsupp2-data1.zip › Figure 1-figure supplement 2A-source data 1.pdf]

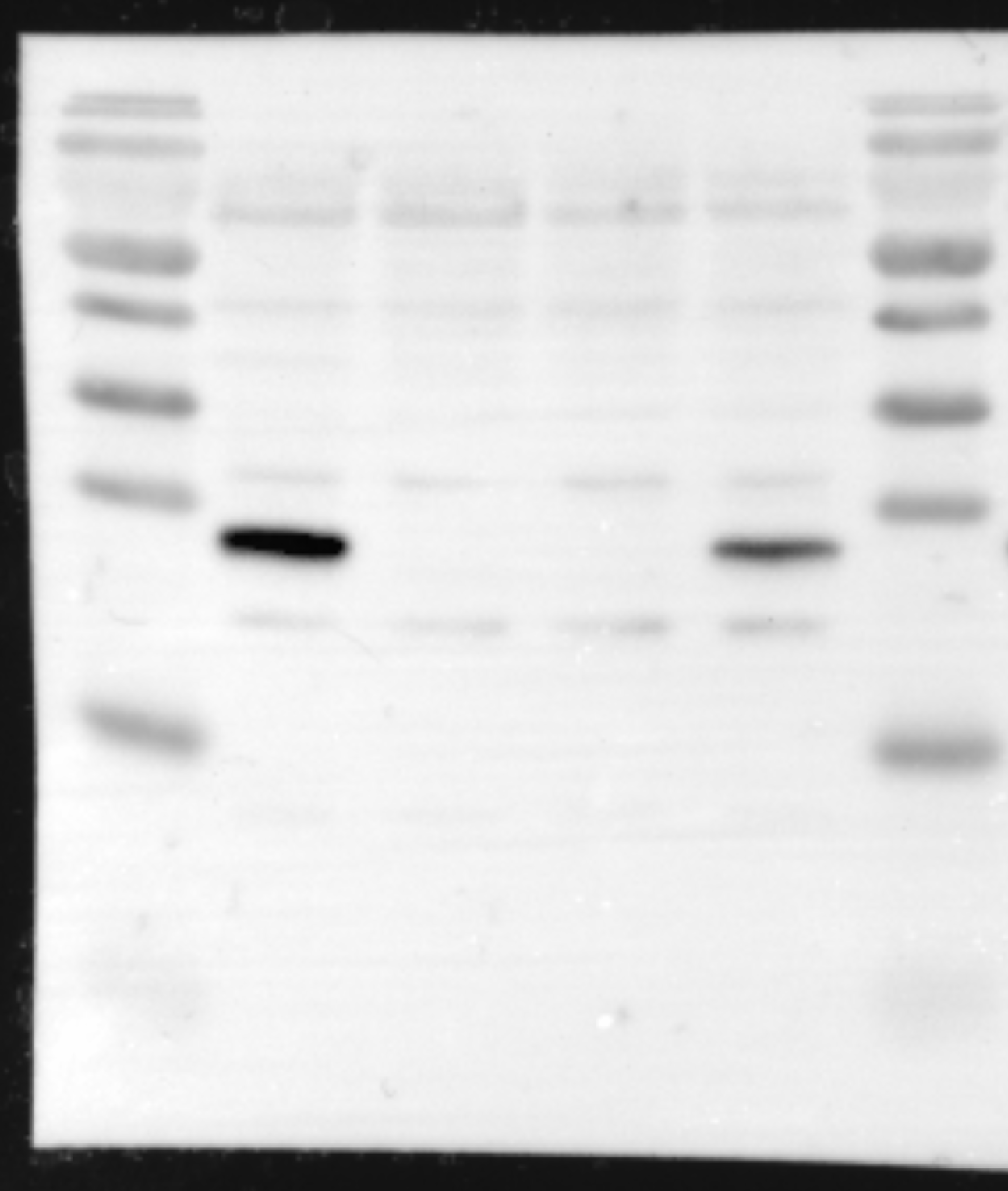

Supplement: Figure 1—figure supplement 2—source data 1. [file elife-86577-fig1-figsupp2-data1.zip › Figure 1-figure supplement 2A-source data 1.tif]

Wild type  
 $\Delta bacA$

Time [h] of BacA depletion

0 1 2 3 4 5 6 7 12 24

kDa

25

15

← BacA

anti-BacA

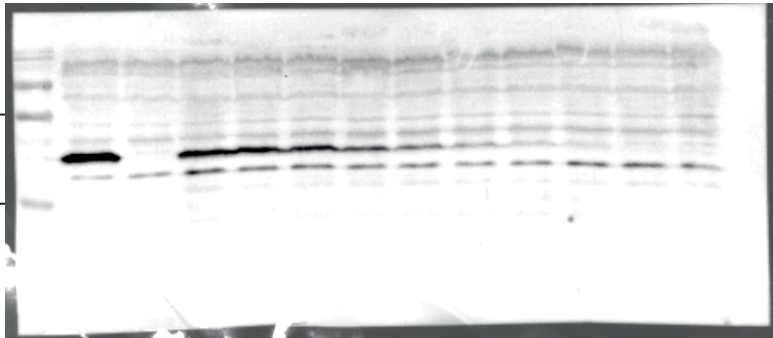

Supplement: Figure 2—source data 1. [file elife-86577-fig2-data1.zip › Figure 2A-source data 1.pdf]

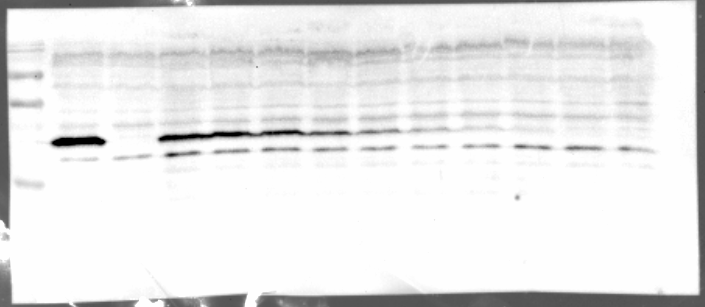

Supplement: Figure 2—source data 1. [file elife-86577-fig2-data1.zip › Figure 2A-source data 1.tif]

Wild type  
BacA-YFP  
BacA<sub>F130R</sub>-YFP

kDa

70

55

35

25

15

10

anti-GFP

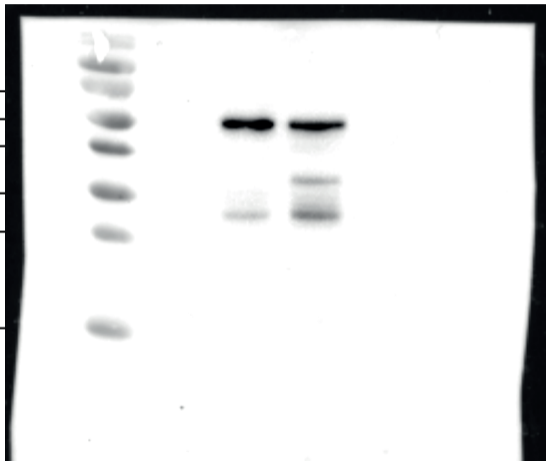

Supplement: Figure 4—figure supplement 1—source data 1. [file elife-86577-fig4-figsupp1-data1.zip › Figure 4-figure supplement 1A-source data 1.pdf]

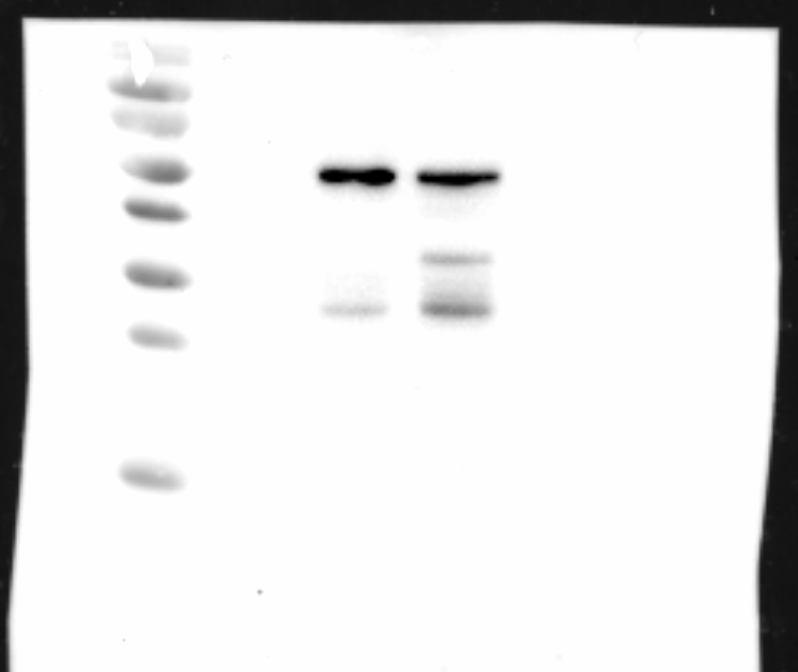

Supplement: Figure 4—figure supplement 1—source data 1. [file elife-86577-fig4-figsupp1-data1.zip › Figure 4-figure supplement 1A-source data 1.tif]

WT

BacD-Venus

kDa

70

55

35

25

15

10

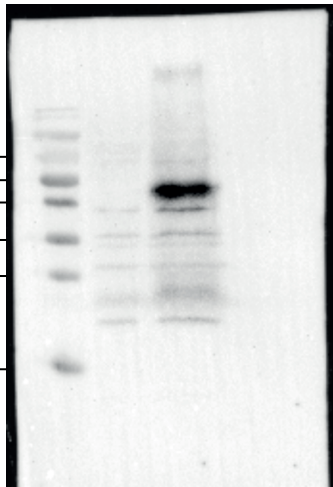

anti-GFP

Supplement: Figure 4—figure supplement 1—source data 1. [file elife-86577-fig4-figsupp1-data1.zip › Figure 4-figure supplement 1B-source data 1.pdf]

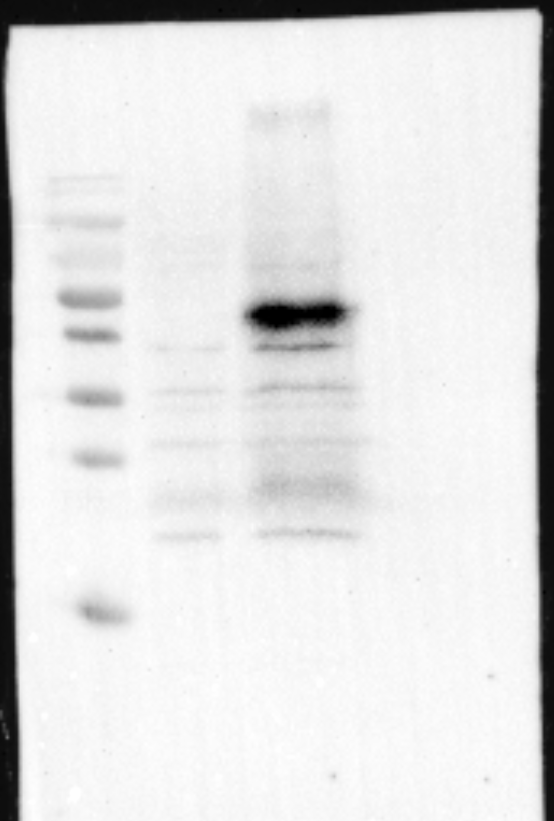

Supplement: Figure 4—figure supplement 1—source data 1. [file elife-86577-fig4-figsupp1-data1.zip › Figure 4-figure supplement 1B-source data 1.tif]

|    |      |              |                    |        |   |              |   |
|----|------|--------------|--------------------|--------|---|--------------|---|
| WT | SU34 | sgLmdC (pre) | Empty vector (pre) | sgLmdC |   | Empty vector |   |
|    |      |              |                    | -      | + | -            | + |

kDa

70

55

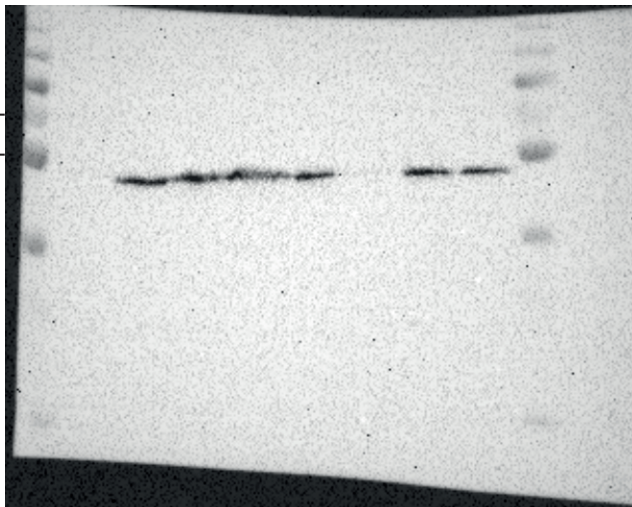

anti-HA

Supplement: Figure 9—source data 1. [file elife-86577-fig9-data1.zip › Figure 9D-source data 1.pdf]

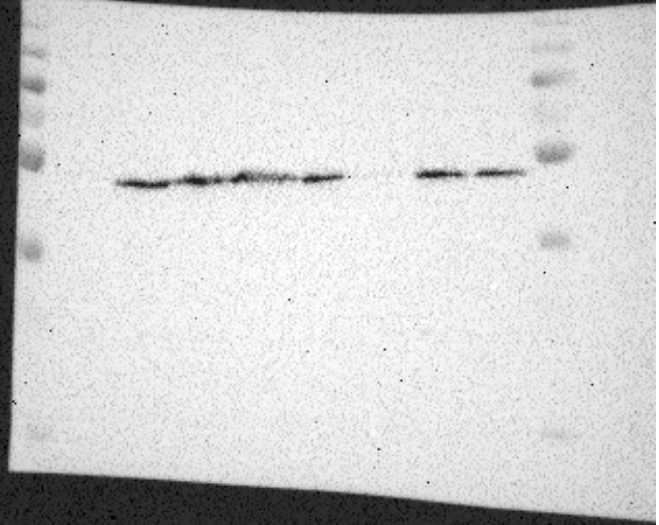

Supplement: Figure 9—source data 1. [file elife-86577-fig9-data1.zip › Figure 9D-source data 1.tif]

WT    BacA-mNG    LmdC<sup>N</sup>-mNG  
                              SP114    SP118    SP119

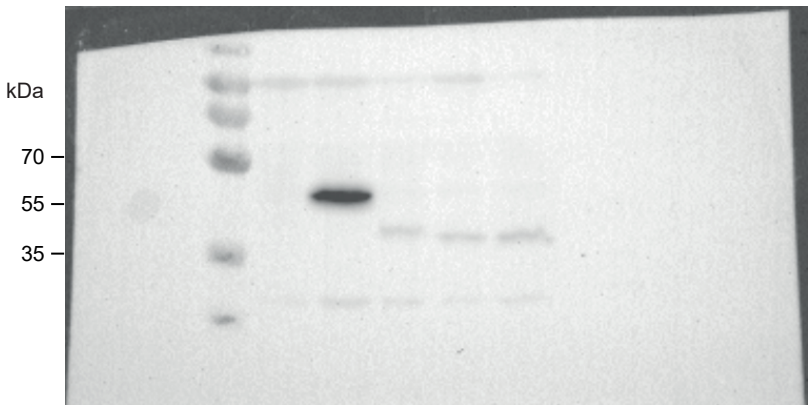

anti-mNeonGreen

Supplement: Figure 10—figure supplement 4—source data 1. [file elife-86577-fig10-figsupp4-data1.zip › Figure 10-figure supplement 4B-source data 1.pdf]

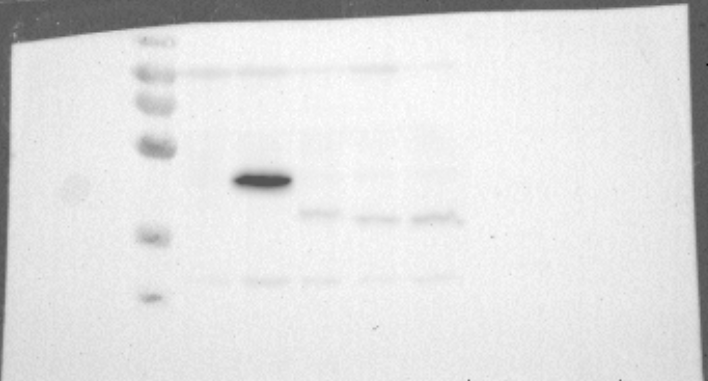

Supplement: Figure 10—figure supplement 4—source data 1. [file elife-86577-fig10-figsupp4-data1.zip › Figure 10-figure supplement 4B-source data 1.tif]

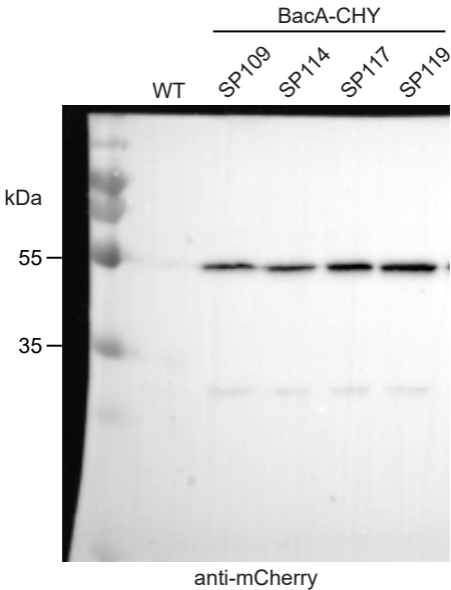

Supplement: Figure 10—figure supplement 4—source data 1. [file elife-86577-fig10-figsupp4-data1.zip › Figure 10-figure supplement 4C-source data 1.pdf]

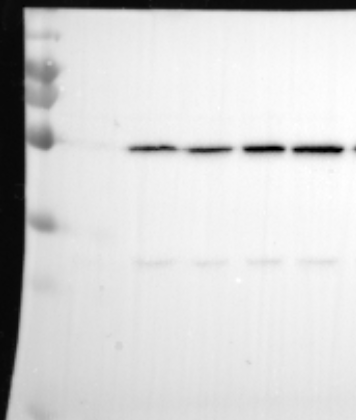

Supplement: Figure 10—figure supplement 4—source data 1. [file elife-86577-fig10-figsupp4-data1.zip › Figure 10-figure supplement 4C-source data 1.tif]
