# Supplementary material for: A dynamic bactofilin cytoskeleton cooperates with an M23 endopeptidase to control bacterial morphogenesis
Source: eLife. 2024 Jan 31;12:RP86577. doi: 10.7554/eLife.86577 (PMC10945521; doi:10.7554/eLife.86577)
Supplement: Supplementary file 3. [file elife-86577-supp3.docx]

**Supplementary file 3**

**Supplementary file 3A. Strains used in this study.**

| **Strain** | **Genotype/description** | **Construction** | **Reference/Source** |
| --- | --- | --- | --- |
|  | |  |  |
| ***H. neptunium*** | |  |  |
| LE670 | Wild type (*aka* ATCC 15444) | - | Leifson, 1964 |
| EC23 | ∆*HNE_0444* (*bacD*) | In-frame deletion of *bacD* in  ATCC 15444 using pEC29 | This study |
| EC28 | ∆*HNE_2629* (*bacA*) | In-frame deletion of *bacA* in  ATCC 15444 using pEC32 | This study |
| EC33 | ∆*bacA* ∆*bacD* | In-frame deletion of *bacD* in  EC28 using pEC29 | This study |
| EC41 | ∆*bacA* P_Cu_::P_Cu_‐*bacA* | Integration of pEC60 in EC28 | This study |
| EC43 | ∆*bacA* P_Zn_::P_Zn_‐*bacD* | Integration of pEC61 into EC28 | This study |
| EC60 | ∆*bacA* P_Zn_::P_Zn_‐*bacD-venus* | Integration of pEC59 into EC28 | This study |
| EC61 | *bacA::bacA*-*eyfp* | Replacement of *bacA* with *bacA-eyfp* in ATCC 15444 using pEC74 | This study |
| EC67 | *bacD::bacD-venus* | Replacement of *bacD* with *bacD-venus* in ATCC 15444 using pEC75 | This study |
| EC68 | *bacA::bacA*-*eyfp bacD::bacD-mCherry* | Replacement of *bacD* with *bacD-mCherry* in EC61 using pEC76 | This study |
| EC93 | *HNE_0620*::e*yfp-HNE_0620* (*rodZ*) | Replacement of *rodZ* with *eyfp-rodZ* in ATCC 15444 using pEC129 | This study |
| MO78 | *bacA*::*bacA*_F130R_-*eyfp* | Replacement of *bacA* with *bacA_F130R_-eyfp* in ATCC 15444 using pMO93 | This study |
| SP221 | ∆*bacA* ∆*bacD rodZ*::*eyfp-RodZ* | Replacement of *rodZ* with *eyfp-rodZ* in EC33 using pEC129 | This study |
| SP236 | *lmdC*::*lmdC*_AA1-65_*-HA-lmdC*_AA66-405_ pdCas9Entry-sgLmdC | Integration of pJH13 in SU34 | This study |
| SP249 | *lmdC*::*lmdC*_AA1-65_*-HA-lmdC*_AA66-405_ pdCas9Entry | Integration of pdCas9Entry in SU34 | This study |
| SU34 | *lmdC*::*lmdC*_AA1-65_*-HA-lmdC*_AA66-405_ | Replacement of *lmdC* with *lmdC*_AA1-65_*-HA-lmdC*_AA66-405_ in  ATCC 15444 using pSU23 | This study |
| ***R. rubrum*** | |  |  |
| S1 | wild type (aka ATCC 11170 or DSM467) |  | Molisch, 1907 |
| SP68 | ∆Rru_A1868 (*lmdC*) | In-frame deletion of *lmdC* in S1 using pSP81 | This study |
| SP70 | ∆Rru_A1867 (*bacA*) | In-frame deletion of *bacA* in S1 using pSP82 | This study |
| SP98 | ∆*lmdC bacA::bacA-mNeongreen* | Replacement of *bacA* with *bacA-mNeongreen* in SP68  using pSP119 | This study |
| SP105 | ∆*bacA* P*_bacA_*-*bacA* | Transformation of SP70 with pSP118 | This study |
| SP109 | *bacA*::*bacA-mCherry* | Replacement of *bacA* with *bacA-mCherry* in S1  using pSP117 | This study |
| SP114 | *bacA*::*bacA-mCherry* P_lmdC_-*lmdC_1-80_-mNeongreen* | Transformation of SP109 with pSP112 | This study |
| SP116 | ∆*bacA* ∆*lmdC* | In-frame deletion of *lmdC* in SP70 using pSP81 | This study |
| SP117 | *bacA*::*bacA-mCherry* ∆*lmdC* | In-frame deletion of *lmdC* in SP109 using pSP130 | This study |
| SP118 | ∆*bacA* ∆*lmdC* P_lmdC_-*lmdC**_1-80_-mNeongreen* | Transformation of SP116 with pSP112 | This study |
| SP119 | *bacA*::*bacA-mCherry* ∆*lmdC* P_lmdC_-*lmdC_1-80_-mNeongreen* | Transformation of SP117 with pSP112 | This study |
| SP237 | *bacA*::*bacA-mCherry* Δ*lmdC* P*_lmdC_-lmdC*_AA1-80_*_;_* _R25A H26A L27A_*-mNeongreen* | Integration of pSP198 in SP117 | This study |
| SP238 | *bacA*::*bacA-mCherry* Δ*lmdC* P*_lmdC_-lmdC*_AA1-80_*_;_* _R30A S31A_*-mNeongreen* | Integration of pSP199 in SP117 | This study |
| ***E. coli*** | |  |  |
| BL21(DE3) | *E. coli* B *dcm* *ompT* *hsdS*(rB^-^ mB^-^) *gal* |  | Invitrogen |
| Rosetta(DE3)pLysS | F^-^ *ompT hsdS*_B_(r_B_^-^ m_B_^-^) *gal dcm* (DE3) pLysSRARE (Cam^R^) | - | Merck Millipore |
| TOP10 | F^–^ mcrA Δ(mrr-hsdRMS-mcrBC) Φ80lacZΔM15 ΔlacX74 recA1 araD139 Δ(ara leu) 7697 galU galK rpsL (Str^R^) endA1 nupG | - | Thermo Fisher Scientific |
| WM3064 | *thrB1004 pro thi rpsL hsdS lacZ*ΔM15 RP4–1360 Δ(*araBAD*)567 Δ*dapA*1341::[*erm pir*(wt)] | - | W. Metcalf (unpublished) |
|  |  |  |  |

**Supplementary file 3B. Backbone plasmids used in this work.**

| **Plasmid** | **Description** | **Source** |
| --- | --- | --- |
|  |  |  |
| pCCFPC-3 | Integrating plasmid for generation of C‐terminal CFP fusions under control of P_Cu_, Rif^R^ | Jung et al., 2015 |
| pCCHYC-2 | Integrating plasmid for generation of C‐terminal mCherry fusions under control of P_Cu_, Kan^R^ | Jung et al., 2015 |
| pCCHYC-3 | Integrating plasmid for generation of C‐terminal mCherry fusions under control of P_Cu_, Rif^R^ | Jung et al., 2015 |
| pCCHYN-2 | Integrating plasmid for generation of N‐terminal mCherry fusions under control of P_Cu_, Kan^R^ | Jung et al., 2015 |
| pCVENC-3 | Integrating plasmid for generation of C‐terminal Venus fusions under control of P_Cu_, Rif^R^ | Jung et al., 2015 |
| pdCas9-humanized | Plasmid carrying a codon-optimized version of the *dCas9* gene | Qi et al., 2013 |
| pET21a(+) | Plasmid for overexpression of C‐terminally His6‐tagged proteins, Amp^R^ | Novagen |
| pNPTS138 | *sacB*‐containing suicide vector used for double homologous recombination, Kan^R^ | M. R. K. Alley, unpublished |
| pRSFDuet-1 | Plasmid for the coexpression of genes under the control of the T7 promoter | Novagen |
| pRXMCS-2 | Low-copy replicative plasmid for ectopic expression of genes under control of P_xyl_ , Kan^R^ | Thanbichler et al., 2007 |
| pTB146 | Plasmid for overexpression of N-terminally His6-SUMO-tagged proteins, Amp^R^ | T. Bernhard (unpublished) |
| pXYFPC-2 | Integrating plasmid for generation of C‐terminal eYFP fusions under control of P_xyl_, Kan^R^ | Thanbichler et al., 2007 |
| pZVENC-2 | Integrating plasmid for generation of C‐terminal Venus fusions under control of P_Zn_, Kan^R^ | Jung et al., 2015 |
|  |  |  |

**Supplementary file 3C. Plasmids generated in this work.**

| **Plasmid** | **Description** | **Construction** |
| --- | --- | --- |
|  |  |  |
| pdCas9Entry | Plasmid carrying (i) humanized *dCas9* under the control of P_Cu_ and (ii) an sgRNA expression cassette comprising the strong constitutive P_HNE_0038_ promoter followed by a BbsI restriction site, a gene fragment encoding the Cas9 sgRNA handle region and a transcriptional terminator. | a) amplification of a synthetic DNA fragment containing P_HNE_0038_, a BbsI restriction site, a gene fragment encoding the Cas9 sgRNA handle region and a transcriptional terminator (Integrated DNA Technologies, USA) using primers oJH19 and oJH20  b) Gibson assembly of the resulting fragment with NheI-treated pJH01 |
| pEC29 | pNPTS138 derivative for in-frame deletion of *bacD* (*HNE_0444*) | a) amplification of *HNE_0444* upstream and downstream regions from *H. neptunium* chromosomal DNA using the primer pairs HNE_0444_del1/2 and HNE_0444_del3/4  b) restriction of the upstream fragment with EcoRI and HindIII and of the downstream fragment with HindIII and NheI  c) triple ligation with pNPTS138 cut with EcoRI and NheI |
| pEC32 | pNPTS138 derivative for in-frame deletion of *bacA* (∆*HNE_2629*) | a) amplification of the *HNE_2629* upstream and downstream regions from *H. neptunium* chromosomal DNA using primer pairs HNE_2629_del1/2 new and HNE_2629_del3/4  b) restriction of the upstream fragment with EcoRI and HindIII and of the downstream fragment with EcoRI and NheI  c) triple ligation with pNPTS138 cut with HindIII and NheI |
| pEC60 | pCVENC-3 carrying *bacA* | a) amplification of *HNE_2629* from *H. neptunium* chromosomal DNA using primers HNE_2629_for and HNE_2629_comp.rev  b) restriction of the PCR product with NdeI and KpnI  c) ligation with pCVENC-3 cut with NdeI and KpnI |
| pEC59 | pZVENC-2 harbouring *bacD* | a) amplification of *HNE_0444* from *H. neptunium* chromosomal DNA using primers HNE_0444_for and HNE_0444_rev  b) restriction of the PCR product with NdeI and KpnI  c) ligation with pZVENC-2 cut with NdeI and KpnI |
| pEC74 | pNPTS138 derivative for the replacement of *bacA* with *bacA-eYFP* | a) amplification of an *HNE_2629-yfp* fragment from pSW56 using primers HNE_2629_HA_for and HNE_2629-FP_eol_rev  b) amplification of the  *HNE_2629* downstream region from *H. neptunium* chromosomal DNA using primers HNE_2629-FP_eol_for and HNE_2629_del4  c) fusion of the two fragments by overlap extension PCR using primers HNE_2629_HA_for and HNE_2629_del4 d) restriction of the resulting PCR fragment with HindIII and NheI and ligation with pNPTS138 cut with HindIII and NheI |
| pEC75 | pNPTS138 derivative for the replacement of *bacD* with *bacD*-*venus* | a) amplification of an *HNE_0444-venus* fragment from pEC59 using primers HNE_0444_for and HNE_0444-FP_eol_rev  b) amplification of the *HNE_0444* downstream region from *H. neptunium* chromosomal DNA using primers HNE_0444-FP_eol_for and HNE_0444_integ_rev  c) fusion of the two fragments by overlap extension PCR using primers HNE_0444_for2 and HNE_0444_del1  d) amplification of a DNA fragment containing *HNE_0444-venus* and the  *HNE_0444* downstream region from the resulting PCR product using primers HNE_0444_for3 and HNE_0444_del1extra  e) restriction of the PCR fragment with HindIII and NheI and ligation with pNPTS138 cut with HindIII and NheI |
| pEC76 | pNPTS138 derivative for the replacement of *bacD* with *bacD-mCherry* | a) amplification of an *HNE_0444-mCherry* fragment from pEC94 using primers HNE_0444_for2 and HNE_0444-FP_eol_rev  b) amplification of the *HNE_0444* downstream region from *H. neptunium* chromosomal DNA using primers HNE_0444FP_eol_for and HNE_0444_del1  c) fusion of the two PCR fragments by overlap extension PCR using primers HNE_0444_for3 and HNE_0444_del1  d) ) amplification of a DNA fragment containing *HNE_0444-mCherry* and the  *HNE_0444* downstream region using primers HNE_0444_for3 and HNE_0444_del1extra  e) restriction of the resulting PCR fragment with HindIII and NheI and ligation with pNPTS138 cut with HindIII and NheI |
| pEC86 | pET21a(+) carrying *bacA* | a) amplification of *HNE_2629* from *H. neptunium* chromosomal DNA using primers HNE_2629*_*for and HNE_2629_rev  b) restriction of the PCR product with EcoRI and NdeI  c) ligation with pET21a (+) cut with EcoRI and NdeI |
| pEC94 | pCCHYC-3 carrying *bacD* | a) amplification of *HNE_0444* from *H. neptunium* chromosomal DNA using primers HNE_0444_for and HNE_0444_rev  b) restriction with of the PCR product with NdeI and KpnI  c) ligation with pCCHYC-3 cut with NdeI and KpnI |
|  |  |  |

**Supplementary file 3C. Plasmids generated in this work (continued).**

| **Plasmid** | **Description** | **Construction** |
| --- | --- | --- |
| pEC119 | pRSFDuet-1 carrying P_T7_-*bacA-eyfp* | a) amplification of *bacA-yfp* from pEC74 using primers mCherry/venus_rev and NE_2629_for3  b) restriction with of the PCR product with PciI and BamHI  c) ligation into pRSFDuet-1 cut with NcoI and BamHI |
| pEC120 | pRSFDuet-1 carrying P_T7_-*bacD-ecfp* | a) amplification of *bacD* from *H. neptunium* chromosomal DNA using primers HNE_0444_for and HNE_0444_rev  b) restriction of the PCR product with NdeI and KpnI  c) ligation into pCCFPC-3 cut with NdeI and KpnI (resulting in pEC70)  d) amplification of *bacD-cfp* from pEC70 using primers HNE_0444_for and ecfp_rev2  e) restriction of the PCR product with NdeI and MfeI  f) ligation into pRSFDuet-1 cut with NdeI and MfeI |
| pEC121 | pRSFDuet-1 carrying P_T7_-*bacA-eyfp* P_T7_-*bacD-ecfp* | a) amplification of *bacB-cfp* using primers HNE_0444_for and ecfp_rev2  b) restriction of the PCR product with NdeI and MfeI c) ligation into pEC119 cut with NdeI and MfeI |
| pEC129 | pNPTS138 derivative for the replacement of *rodZ* with *eyfp-rodZ* | a) amplification of *eyfp* from pXYFPC-2 using primers HNE_0620_eol_for2 and HNE_0620_eol_rev2  b) amplification of the regions flanking the *eyfp* integration site from *H. neptunium* chromosomal DNA using primer pairs HNE_0620_eol_for/HNE_0620_eol_rev and HNE_0620_eol_for3/HNE_0620_eol_rev3  c) fusion of the three PCR fragments by overlap extension PCR using primers HNE_0620_eol_for and HNE_0620_eol_rev3  d) restriction of the PCR product with HindIII and NheI and ligation with pNPTS138 cut with HindIII and NheI |
| pJH01 | pCCHYN-2 carrying a codon-optimized version of *dCas9* | a) PCR amplification of *dCas9* from pdCas9-humanized using primers oJH13 and oJH14  b) Fusion of the PCR fragment with NdeI/KpnI-treated pCCHYN-2 using Gibson assembly |
| pJH13 | pdCas9Entry carrying an sgRNA targeting *lmdC* | a) phosphorylation and subsequent annealing of oligonucleotides oJH48 and oJH4  b) ligation with pdCas9Entry cut with BbsI |
| pMO93 | pNPTS138 derivative for the replacement of *bacA* with *bacA_F130R_-eYFP* | Site-directed mutagenesis of pEC74 with primers bacA-Hn-F130R-for and bacA-Hn-F130R-rev |
| pSP81 | pNPTS138 derivative for in-frame deletion of *lmdC_Rs_* (∆*Rru_A1868*) | a) amplification of the upstream and downstream regions of *Rru_A1868* from *R. rubrum* chromosomal DNA using the primer pairs oSP309/oSP310 and oSP311/oSP312  b) insertion of the two fragments into pNPTS138 cut with HindIII and NheI by Gibson assembly |
| pSP82 | pNPTS138 derivative for in-frame deletion of *bacA_Rs_* (∆*Rru_A1867*) | a) amplification of the upstream and downstream regions of *Rru_A1867* from *R. rubrum* chromosomal DNA using the primer pairs oSP315/oSP316 and oSP317/oSP318  b) insertion of the two PCR products into pNPTS138 cut with HindIII and NheI by Gibson assembly |
| pSP112 | pRXMCS-2 carrying P*_lmdC_*-*lmdC_1-80_-mNeongreen* | a) amplification of P*_lmdC_*-*lmdC_(AA1-80)_* from *R. rubrum* chromosomal DNA using primers oSP394 and oSP435  b) amplification of a fragment encoding a linker and mNeongreen using primers oSP436 and oSP218  c) insertion of the two PCR products into pRXMCS-2 cut with NotI and EcoRI by Gibson assembly |
| pSP117 | pNPTS138 derivative for the replacement of *bacA* with *bacA-mCherry* | a) amplification of the regions flanking the *mCherry* integration site from *R. rubrum* chromosomal DNA using the primer pairs oSP321/oSP322 and oSP464/oSP318  b) amplification of a fragment encoding a linker and mCherry using primers oSP323 and oSP463  c) insertion of the two PCR products into pNPTS138 cut with HindIII and NheI by Gibson assembly |
| pSP118 | pRXMCS-2 carrying P*_lmdC_*-*bacA_Rs_* | a) amplification of P*_lmdC_* and *bacA* from *R. rubrum* chromosomal DNA using the primer pairs oSP394/oSP465 and oSP466/oSP467  b) insertion of the two PCR products into pRXMCS-2 cut with NotI and EcoRI by Gibson assembly |
| pSP119 | pNPTS138 derivative for the replacement of *bacA* with *bacA-mNeongreen* in the ∆*lmdC* background | a) amplification of the regions flanking the *mNeongreen* integration site from SP68 chromosomal DNA using the primer pairs oSP468/oSP322 and oSP325/oSP318  b) amplification of a fragment encoding a linker and mNeongreen using primers oSP323 and oSP324  c) insertion of the two PCR products into pNPTS138 cut with HindIII and NheI by Gibson assembly |
| pSP120 | pET21a(+) carrying *lmdC_Rs_* | a) amplification of *Rru_A1868* from *R. rubrum* chromosomal DNA using primers oSP469 and oSP470  b) insertion of the PCR product into pET21a(+) cut with HindIII and NdeI by Gibson assembly |
| pSP130 | pNPTS138 derivative for in-frame deletion of *lmdC_Rs_* (∆*Rru_A1868*) in the *bacA_Rs_::bacA_Rs_-mCherry* background | a) amplification of the regions flanking *Rru_A1868* from SP109 chromosomal DNA using the primer pairs oSP309/oSP310 and oSP311/oSP485  b) insertion of the PCR products into pNPTS138 cut with HindIII and NheI by Gibson assembly |
|  |  |  |

**Supplementary file 3C. Plasmids generated in this work (continued).**

| **Plasmid** | **Description** | **Construction** |
| --- | --- | --- |
| pSP198 | pSP112 carrying P*_lmdC_*-*lmdC_1-80_*  *_R25A H26A L27A_ -mNeongreen* | a) Site-directed mutagenesis of pSP112 with primers oSP690 and oSP691 |
| pSP199 | pSP112 carrying P*_lmdC_*-*lmdC_1-80_*  *_R30A S31A_ -mNeongreen* | a) Site-directed mutagenesis of pSP112 with primers oSP692 and oSP693 |
| pSU23 | pNPTS138 derivative for the replacement of *lmdC* with  *lmdC*::*lmdC*_AA1-65_*-HA-lmdC*_AA66-405_ | a) amplification of the regions upstream and downstream of the *HA* integration site from *H. neptunium* chromosomal DNA using the primer pairs oSU36/oSU41 (fragment 1) and oSU39/oSU42 (fragment2)  b) amplification of the upstream region from fragment 1 with primers oSU38 and oSU43 (fragment 1.1)  c) insertion of fragments 1.1 and 2 into pNPTS138 cut with HindIII and NheI by Gibson assembly |
| pSW56 | pXYFPC-2 carrying *bacA* | a) amplification of *HNE_2629* from *H. neptunium* chromosomal DNA using primers *HNE2629*-for and *HNE2629*-rev  b) ligation with pXYFPC-2 cut with EcoRI and NdeI |
| pYL15 | pTB146 carrying *lmdC*_226-345_ | a) PCR amplification of lmdC with primers lmdC*_M23*_for and *lmdC*_M23_rev  b) Insertion of the *lmdC*_226-345_ fragment into pTB146 cut with BamHI and SapI by  Gibson assembly |

**Supplementary file 3D. PCR primers used in this work.**

| **Oligonucleotide** | **Sequence** |
| --- | --- |
|  |  |
| ecfp_rev2 | tatcaattgttacttgtacagctcgtc |
| HNE_0444_del1 | aaagaattccaggccgaactcgccatcgaaaagg |
| HNE_0444_del2 | tataagcttacagccgtctagttgttctgcagg |
| HNE_0444_del3 | tataagcttatctgctgccatccgcgtgtctccc |
| HNE_0444_del4 | ttttgctagccagttgtgcgcgtgttcgagatcg |
| HNE_0444_for | tatatacatatggcagcagataaggcaagggaaccg |
| HNE_0444_rev | tataggtaccgacggctgtgctggccggcggctc |
| HNE_2629_del1 | tatgaattctcggcggagatcagtccttcatgac |
| HNE_2629_del2 | tataagcttgttattctttgtgaacatgtttgcc |
| HNE_2629_del3 | ttttaagcttccgccgagctgatcgcgcgagggtc |
| HNE_2629_del4 | ttttgctagcacgcgcttgtctgcttcgaggttca |
| HNE_2629_for3 | cgcacatgttcacaaagaataacaaaaccccagc |
| HNE_0444_for | tatatacatatggcagcagataaggcaagggaaccg |
| HNE_0444_rev | tataggtaccgacggctgtgctggccggcggctc |
| HNE_2629_for | ttaacatatgttcacaaagaataacaaaaccccagcggc |
| HNE_2629_rev | tagaattcgagctcggcggcgaggaactcgagatg |
| HNE_2629_comp.rev | tataggtacctcagctcggcggcgaggaactcgag |
| HNE_2629_HA_for | tataagcttatgttcacaaagaataacaaaaccccagc |
| HNE_2629-FP_eol_rev | tcgcgcgatcagctcggcggttacttgtacagctcgtcca |
| HNE_2629-FP_eol_for | tggacgagctgtacaagtaaccgccgagctgatcgcgcga |
| HNE_0444-FP_eol_rev | gtctgcgcaacctgcagaacaattacttgtacagctcgtcca |
| HNE_0444-FP_eol_for | tggacgagctgtacaagtaattgttctgcaggttgcgcagac |
| HNE_0444_for2 | tataagcttatggcagcagataaggcaagggaaccg |
| HNE_0444_for3 | tatagctagcatggcagcagataaggcaagggaaccg |
| HNE_0444_del1extra | tttaagcttcaggccgaactcgccatcgaaaagg |
| HNE_0620_eol_for | tatagctagccgcgctcgaccataaaggt |
| HNE_0620_eol_rev | tcctcgcccttgctcaccatattctaccagtcacttcgac |
| HNE_0620_eol_for2 | gtcgaagtgactggtagaatatggtgagcaagggcgagga |
| HNE_0620_eol_rev2 | tgggtcatgttgtgtgccatatgcatattaattaaggcgc |
| HNE_0620_eol_for3 | gcgccttaattaatatgcatatggcacacaacatgaccca |
| HNE_0620_eol_rev3 | tttaagcttcggccagtgtgcggctgag |
| bacA-Hn-F130R-for | ttcacctggccttcacgcacggcgtttgactgga |
| bacA-Hn-F130R-rev | agtcaaacgccgtgcgtgaaggccaggtgaagcat |
| lmdC_TMH_for | cattcacaggaactcttccatatggcgaagtggagtgcca |
| lmdC_TMH_rev | gccgaccggtgacgcgtaacgttcgcggggccgccgccgacg |
| lmdC_M23_for | gctcacagagaacagattggtggcattcgcgtcgacccct |
| lmdC_M23_rev | gctttgttagcagccggatccttattctttgtgaacatgttt |
| mCherry/venus_rev | ataggatccttacttgtacagctcgtccat |
| oJH13 | acaggaactcttccatatggacaagaagtattctatcggactggccatc |
| oJH14 | cgagatcttaaggtacctcaatcccctccgagctgtgagagg |
| oJH19 | gttacgcgtaccggtggcggccgccatcgtggcgg |
| oJH20 | tcccccgggctgcagctagcaaaaaaagcaccgactcggtgccac |
| oJH48 | accagcgatgatagatctggcgttc |
| oJH49 | aaacgaacgccagatctatcatcgc |
| oSP218 | caccacgtggtacctcgagttacttgtacagctcgtccatgcccatcac |
| oSP309 | gtgcaattgaagccggctggcgccacatgatgccaaagccgccggg |
| oSP310 | catttttccccgctttcaagaaggaaaggtgcagatcctgggggtc |
| oSP311 | ggatctgcacctttccttcttgaaagcggggaaaaatgttttcgaaggc |
| oSP312 | catccggagacgcgtcacggccgaagggccgaccgaatggatcgccc |
| oSP315 | gcaattgaagccggctggcgccaggtgctgctgaccgatttcgatgg |
| oSP316 | cgggttcagggtgccgtcgccggcggtcgaccgtttggagc |
| oSP317 | gctccaaacggtcgaccgccggcgacggcaccctgaaccc |
| oSP318 | catccggagacgcgtcacggccgaagaacaccaagcacaagggggacg |
| oSP321 | gtgcaattgaagccggctggcgccatggccggctgtcgcgc |
| oSP322 | cgcgtaacgttcgaattctccggagctcgggaggccgccgccgg |
| oSP323 | cccggcggcggcctcccgagctccggagaattcgaacgttacg |
| oSP324 | ccggcagtccaggcggcgattacttgtacagctcgtccatgcccatcac |
| oSP325 | gtgatgggcatggacgagctgtacaagtaatcgccgcctggactgccg |
| oSP394 | cagcgagtcagtgagcgaggaagctcgggtccgggcggcac |
| oSP435 | cctcctcgcccttgctcaccatgatgcgttcgttcttggccgacag |
| oSP436 | cggccaagaacgaacgcatcatggtgagcaagggcgaggaggataac |
| oSP463 | cccggcagtccaggcggcgattacttgtacagctcgtccatgccgc |
| oSP464 | gcggcatggacgagctgtacaagtaatcgccgcctggactgccg |
| oSP465 | gtttggagctagccttcgaaaacatgaggtctcctgttccaaatccgcc |
|  |  |

**Supplementary file 3D. PCR primers used in this work (continued)**

| **Oligonucleotide** | **Sequence** |
| --- | --- |
|  |  |
| oSP466 | gatttggaacaggagacctcatgttttcgaaggctagctccaaacggtc |
| oSP467 | cttaagagctcaccacgtggtacctcgagtcaggaggccgccgccg |
| oSP468 | caattgaagccggctggcgccaatttttttgttgtatcacgtcaaggcg |
| oSP469 | gtttaactttaagaaggagatatacatatgtcggagttcgacccccagg |
| oSP470 | ggtgctcgagtgcggccgcaagcttgccttcgaaaacatttttccccgc |
| oSP485 | ccggagacgcgtcacggccgaagcccacttgaagccctcggggaag |
| oSP690  oSP691  oSP692  oSP693 | gccgccatttccccgatgcggcggcgatggtccgctccgatggg  cccatcggagcggaccatcgccgccgcatcggggaaatggcggc  cgatcgccacctcatggtcgcggcggatggggcgatgcggc  gccgcatcgccccatccgccgcgaccatgaggtggcgatcg |
| oSU36 | cgggccgccgatgttcgtctgt |
| oSU38 | ggagacgcgtcacggccgaagcgggccgccgatgttcgtc |
| oSU39 | ttgaagccggctggcgccacgcggcgaacctttctgatc |
| oSU41 | tacccatacgacgtcccagactacgctcccggcacagcgcagg |
| oSU42 | tctgggacgtcgtatgggtagccgccgccgacgac |
| oSU43 | aaatttcgtcgtcggcggcggctacccatacgacgtcccagact |
|  |  |

**Supplementary references**

Jung A, Eisheuer S, Cserti E, Leicht O, Strobel W, Möll A, Schlimpert S, Kühn J, Thanbichler M (2015) Molecular toolbox for genetic manipulation of the stalked budding bacterium *Hyphomonas neptunium*. *Appl Environ Microbiol* **81**:736-744.

Leifson E (1964) *Hyphomicrobium neptunium sp. n*. *Antonie Van Leeuwenhoek* **30**:249-256.

Molisch H (1907) Die Purpurbakterien nach neuen Untersuchungen (Jena: Gustav Fischer).

Thanbichler M, Iniesta AA, Shapiro L (2007) A comprehensive set of plasmids for vanillate- and xylose-inducible gene expression in *Caulobacter crescentus*. *Nucleic Acids Res* **35**:e137.

Qi LS, Larson MH, Gilbert LA, Doudna JA, Weissman JS, Arkin AP, Lim WA (2013) Repurposing CRISPR as an RNA-guided platform for sequence-specific control of gene expression. *Cell* **152**: 1173-1183.
